# A Modified Johnson Cook Model-Based Kalman Filter Method to Determine the Hot Flow Behavior of Sustainable AA6082 Al Alloy

**DOI:** 10.3390/ma17215169

**Published:** 2024-10-23

**Authors:** Bandar Alzahrani, Ali Abd El-Aty, Sherif A. Elatriby, Arafa S. Sobh, Mohamed A. Bhlol, Abdullah A. Elfar, Muhammad Ali Siddiqui, Abdallah Shokry

**Affiliations:** 1 Department of Mechanical Engineering, College of Engineering at Al Kharj, Prince Sattam Bin Abdulaziz University, Al Kharj 11942, Saudi Arabia; 2 Mechanical Engineering Department, Faculty of Engineering, Helwan University, Cairo 11795, Egypt; 3 School of Engineering and Applied Science, Smart Engineering Research Centre, Nile University, Giza 12677, Egypt; 4 Computational and Experimental Materials Innovation Group (CEMIG), Department of Metallurgical Engineering, NED University of Engineering and Technology, Karachi 75270, Pakistan; 5 Department of Mechanical Engineering, Faculty of Engineering, Fayoum University, Fayoum 63514, Egypt

**Keywords:** AA6082 alloy, SDGs, SDG 9, flow behavior, strain rate, JC model, elevated temperature, Kalman filter method

## Abstract

AA6082 alloys play a significant role in advancing sustainable development goals (SDGs) by contributing to environmental sustainability, economic growth, and social well-being. These alloys are highly recyclable and align with SDG 12 by promoting resource efficiency and reducing waste. Their application in lightweight vehicles and improving energy efficiency in construction supports SDG 9 and SDG 11, as they help reduce carbon emissions and enhance the sustainability of urban environments. While AA6082 alloys offer significant advantages, their use has limitations that can hinder their industrial applications. One key challenge is their lower formability, particularly at room temperature. Elevated-temperature deformation is frequently employed to enhance the formability of these alloys and address their limitations. Thus, a deep understanding of the constitutive analysis of these alloys under a wide range of T and $\dot{\varepsilon }$ is essential for manufacturing sound components from these alloys. Thus, this study aims to propose a new modification for the JC model (PJCM) and compare its reliability to predict the warm/hot flow behavior of AA6082 alloys with that of the original JC model (OJCM) and the modified JC model (LMJCM). By comparing the experimental results with these model results and confirming the determining correlation coefficient (R), average absolute relative error (AARE), and root mean square error (RMSE) values, it is concluded that the stresses predicted by the PMJCM closely match the experimental stresses of the LMJCM and OJCM because of the interaction between $\dot{\varepsilon }$, ε, and T, which might be a reason for the complex nonlinear behavior of AA6082 alloys during hot deformation.

## 1. Introduction

Sustainable Al alloys minimize environmental impact throughout their lifecycle, from production to end-of-life recycling [1]. These alloys leverage the high recyclability of Al, significantly reducing energy requirements compared with primary production and lowering greenhouse gas emissions [2]. By incorporating eco-friendly alloying elements and optimizing manufacturing processes, sustainable Al alloys reduce material waste and increase energy efficiency [3]. The development of high-strength, lightweight alloys has increased material efficiency in the automotive and aerospace industries, improving fuel economy and reducing the carbon footprint [4,5]. This leads Al alloys to play a significant role in advancing SDGs by contributing to environmental sustainability, economic growth, and social well-being [6]. These alloys are highly recyclable and align with SDG 12 (Responsible Consumption and Production) by promoting resource efficiency and reducing waste [7]. Their applications in lightweight vehicles and in improving energy efficiency in construction supports SDG 9 (Industry, Innovation, and Infrastructure) and SDG 11 (Sustainable Cities and Communities), as they help reduce carbon emissions and enhance the sustainability of urban environments [5,6,7,8,9].

In the modern automobile and aerospace industries, Al-Mg-Si 6xxx series alloys have become increasingly popular for extrusion components because of their unique properties [10]. These alloys offer high specific strength, making them ideal for lightweight structural applications that demand both durability and reduced weight [11]. Their outstanding extrudability allows the manufacture of complex shapes with precise dimensions, which are essential for both automotive body panels and aerospace structural components [12]. Additionally, the excellent weldability of 6xxx alloys ensures that components can be securely joined without compromising their integrity, facilitating the manufacturing of large, intricate assemblies [13]. Moreover, the high recyclability of these alloys aligns with the growing emphasis on sustainability, allowing the production of environmentally friendly components with minimal waste and energy consumption [14]. This trend toward using Al-Mg-Si 6xxx alloys reflects the industry’s commitment to optimizing performance while advancing sustainable manufacturing practices [15].

While Al-Mg-Si 6xxx series alloys offer significant advantages, their use has limitations that can hinder their industrial application [2,16]. One key challenge is their lower formability, particularly at room temperature [17]. This limitation can lead to increased material waste during forming processes, which conflicts with SDG 12 (responsible consumption and production), optimizing material use and reducing scrap [3,18]. Additionally, forming Al-Mg-Si 6xxx alloys requires higher temperatures, necessitating more energy-intensive processes [19,20]. This increased energy demand can result in higher carbon emissions, posing a challenge to SDG 13 (climate action) by contributing to climate change [3].

Alternative techniques, such as high-speed forming and elevated temperature deformation, are frequently employed to increase the formability of Al-Mg-Si 6xxx alloys and address their limitations [21,22,23,24,25,26,27]. Thus, a deep understanding of the deformation behavior of Al-Mg-Si alloys at elevated temperatures is essential for manufacturing sound components from these alloys. Therefore, experimental investigations are needed to evaluate the mechanical behavior of Al-Mg-Si alloys under various temperature (T), and strain rate $\left(\dot{\varepsilon }\right)$, and loading conditions. Additionally, advanced modeling techniques coupled with the finite element method (FEM) should be utilized to simulate and predict the deformation behavior of a material to optimize hot working conditions and to mimic real applications in severe environments [25,26,27,28,29,30,31]. The reliability and accuracy of the FEM in simulating the plastic deformation of metallic materials mainly relies on the constitutive relationships that describe the materials’ behaviors [32,33,34,35]. Several constitutive relationships have been developed and proposed in recent years to predict the warm/hot flow behavior of various metals, including phenomenological, physical, and ANN-based models [35,36,37,38,39,40,41]. An optimal constitutive model should have a moderate number of material parameters, which can be determined via a few experimental data points to accurately predict the flow behavior of metals across a broad range of rheological conditions [41].

Phenomenological models provide a simplified approach for predicting material flow behavior across a wide range of T and $\dot{\varepsilon }$. These models do not require an in-depth understanding of the rheological factors involved in forming technologies [42,43,44,45,46,47]. They are primarily developed through empirical fitting and regression analysis, making them valuable for modeling material flow behavior and integrating with FE codes to simulate several forming technologies under various conditions [42,43,44,45]. Among these models, the Johnson–Cook (JC) model is particularly popular in FE applications because of its quick computation, low computational demands, and straightforward formulation [46,47,48,49,50,51,52,53,54,55,56,57,58,59].

The JC model has been one of the most widely used constitutive models because of its simplicity and practicality in describing the deformation behavior of metals [57]. It is beneficial for predicting large deformations under a wide range of T and $\dot{\varepsilon }$, such as those encountered in industrial processes such as forging, extrusion, and machining [58,59,60]. One of the reasons for its widespread adoption is that the JC model requires fewer material constants than more complex models, making it easier to implement and requiring fewer experimental tests to calibrate [60]. This advantage makes it ideal for modeling the flow stress of metals such as steels [54,61,62,63,64], Al [65,66,67], Mg [68], and even metal matrix composites [69].

However, the JC model also has some inherent limitations. The original formulation assumes that the effects of ε, $\dot{\varepsilon }$, and T on the flow stress are independent of each other. This means that the hardening effects of ε $and\;\dot{\varepsilon },$ as well as the softening effect of T, are considered additive but do not interact. While this assumption simplifies the model, it also limits its accuracy in predicting the actual material behavior, particularly during complex deformation processes where these factors are interdependent. Lin et al. [60] introduced a coupled term that reflects the interaction between these parameters, significantly enhancing the model’s accuracy for high-strength alloys during hot deformation. These modifications are critical for more precise modeling of materials during hot working processes, where dynamic behaviors such as strain hardening, thermal softening, and strain rate sensitivity play important roles [54,58,59,60,61,62,63,64,65,66,67,68,69]. Modified JC models align better with experimental data, particularly in applications requiring FEM to simulate industrial processes [59]. These advancements have made the modified JC model an even more reliable tool for optimizing hot deformation processes, minimizing material defects, and improving manufacturing efficiency [54,60,61,62,63,64,65,66,67,68,69].

One major drawback of the JC model is its inability to simultaneously predict strain hardening and softening behaviors, especially at elevated temperatures [54,60,61,62,63,64,65,66,67,68,69]. Materials exhibit dynamic recrystallization or other softening mechanisms in many hot working processes, which the original JC model does not capture effectively. This can result in significant discrepancies between the predicted and actual flow stresses, particularly in cases where $\dot{\varepsilon }$ and where T interact to produce complex material responses. To overcome these limitations, researchers have proposed several modifications to the original JC model. These modified models aim to incorporate the coupled effects of ε, $\dot{\varepsilon }$, and T, thereby improving the model’s ability to accurately predict flow stress under a wider range of conditions.

Thus, this study aims to propose a new modification for the JC model (PMJCM) and compare its reliability in predicting the warm/hot flow behavior of the AA6082 Al alloy with the original JC model (OJCM) and the modified JC model by Lin et al. [60] (LMJCM). The predictability of these three models is evaluated and compared by calculating the RMSE, AARE, and R.

## 2. Experimental Procedures

In a recently published study, Ghosh et al. [70] investigated the hot deformation behavior and hot workability of an Al-Mg-Si-Zr-Mn alloy (AA6082). To investigate the hot deformation behavior of this alloy, a Gleeble-3800 thermomechanical simulator machine was used with 16 different combinations of $\dot{\varepsilon }$: 0.001 s^−1^, 0.01 s^−1^, 0.1 s^−1^, and 1 s^−1^ and T: 400 °C, 450 °C, 500 °C, and 550 °C. The chemical composition, microstructure, and obtained stress–strain data for the tested alloy with different strain rates and temperatures are found in [70].

The stress–strain curves show that the flow behavior of the AA6082 alloy during hot deformation matches that of most alloys with the same environment of elevated temperatures and different strain rates. The stress increases as the strain rate increases, which might be attributed to the generation and multiplication of dislocations. The generation and multiplication of dislocations play essential roles, since high stresses are required due to dislocation interactions [71,72]. In contrast, stress decreases as the temperature increases, which might be associated with the considerable time required to initiate dynamic recovery and dynamic recrystallization [72,73].

## 3. Constitutive Modeling

In this section, three constitutive models are developed and investigated. The models are the OJCM, the modified JC model by Lin et al. [60] (LMJCM), and a proposed modification for the Johnson–Cook model (PMJCM). Additionally, the determination of the model constants is analyzed and presented. Thus, the comparison between the stresses obtained by the three tested models and the experimental stresses are considered. Furthermore, well-known statistical parameters are implemented to evaluate and assess the tested models.

### 3.1. Original Johnson–Cook Model (OJCM)

In 1983, Johnson and Cook [59] introduced their famous constitutive model to predict the flow behavior of metals and alloys at elevated temperatures and different strain rates. The introduced model contains three independent terms: strain hardening, dynamic recovery, and softening or dynamic recrystallization. The OJCM is given as:

(1) $$\sigma =\left(A+B\varepsilon^{n}\right)\left(1+C\ln{\dot{\varepsilon }}^{*}\right)\left(1-{T^{*}}^{m}\right)$$

where σ represents the flow stress, ε represents the plastic strain, and A, B, n, C, and *m* are material constants. The term ${\dot{\varepsilon }}^{*}=\dot{\varepsilon }/{\dot{\varepsilon }}_{\circ }$ is a dimensionless strain rate in which $\dot{\varepsilon }$ is the tested strain rate and ${\dot{\varepsilon }}_{\circ }$ is a chosen reference strain rate. The term $T^{*}$ introduces a ratio between $T-T_{r}$ and $T_{m}-T_{r}$, in which T and $T_{r}$ are the tested temperatures and a chosen reference temperature, respectively, and $T_{m}$ is the melting temperature.

At a chosen reference temperature of 400 °C and a reference strain rate of 0.001 s^−1^, Equation (1) reduces to:

(2) $$\sigma =A+B\varepsilon^{n}$$

**Figure 1:**
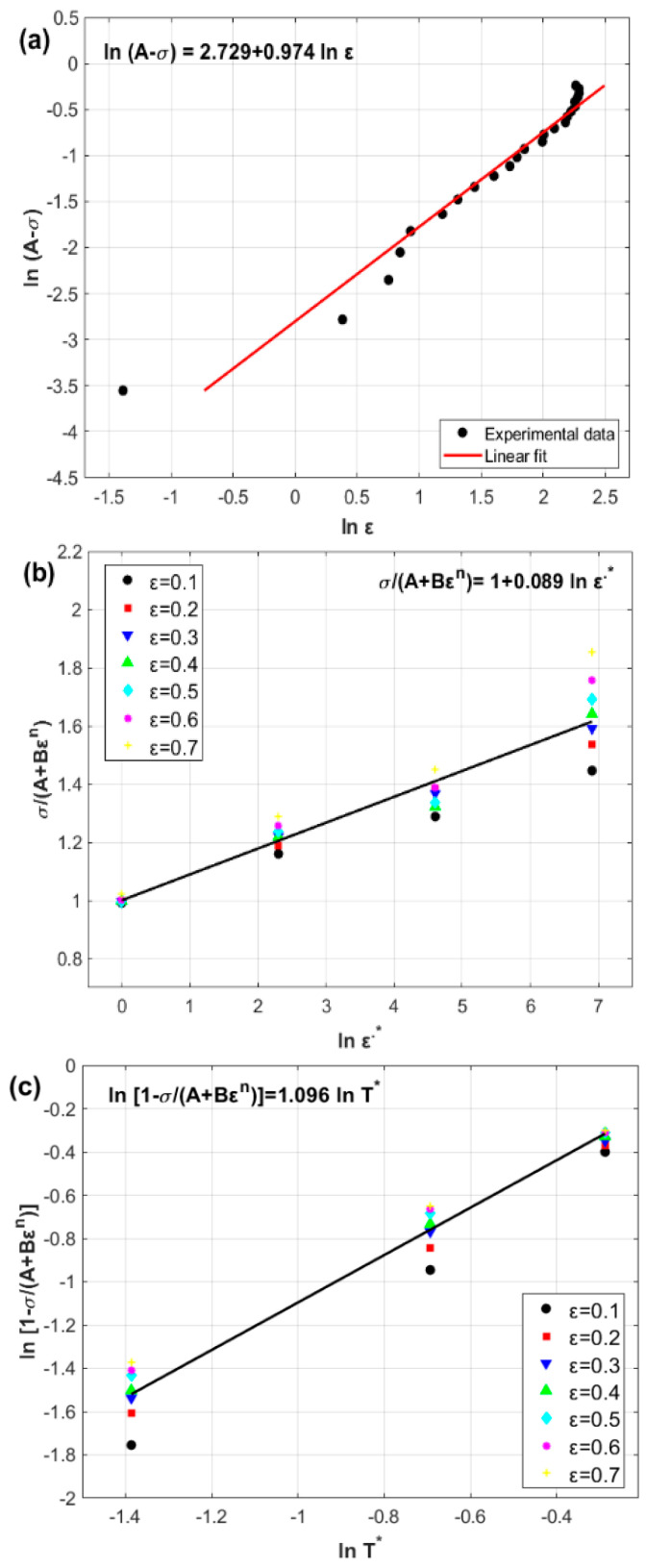
(**a**) Experimental stresses versus strains at reference values of 0.001 s^−1^ and 400 °C, (**b**) $\sigma /\left(A+{B\varepsilon }^{n}\right)$ versus $\ln{\dot{\varepsilon }}^{*}$, and (**c**) $\ln\left[1-\sigma /\left(A+B\varepsilon^{n}\right)\right]$ versus $T^{*}$.

Taking the logarithm after performing several rearrangements, Equation (2) can be linearly introduced as:

(3) ln⁡(A−σ)=ln⁡(−B)+n ln⁡(ε)

where A represents the yield stress of the reference data, which is 58.5 MPa. By plotting ln⁡(A−σ) versus ln⁡(ε), both the constants B and n can be determined from the intercept and slope as −15.31 MPa and 0.974, respectively, as shown in Figure 1a.

At the reference temperature of 400 °C, Equation (1) reduces to:

(4) $$\sigma =\left(A+B\varepsilon^{n}\right)\left(1+C\ln{\dot{\varepsilon }}^{*}\right)$$

After performing some rearrangements, Equation (4) can be linearly introduced as:

(5) $$\frac{\sigma }{A+B\varepsilon^{n}}=1+C\ln{\dot{\varepsilon }}^{*}$$

At strain values ranging from 0.1 to 0.7 with an increment of 0.1, $\sigma /\left(A+B\varepsilon^{n}\right)$ is plotted against $\ln{\dot{\varepsilon }}^{*}$ at different strain rates, in which C is the slope and is determined to be 0.089, as shown in Figure 1b.

At a reference strain rate of 0.001 s^−1^, Equation (1) reduces to:

(6) $$\sigma =\left(A{+B\varepsilon }^{n}\right)\left(1-{T^{*}}^{m}\right)$$

Taking the logarithm and performing some rearrangements, Equation (6) can be written as:

(7) $$\ln\left[1-\frac{\sigma }{A+B\varepsilon^{n}}\right]=m\;T^{*}$$

The constant m is the slope, which is determined to be 1.096 by plotting $\ln\left[1-\sigma /\left(A+B\varepsilon^{n}\right)\right]$ against $T^{*}$, as shown in Figure 1c.

The final OJCM can be expressed as:

(8) $$\sigma =\left(58.5{-15.31\;\varepsilon }^{0.974}\right)\left(1+0.089\ln{\dot{\varepsilon }}^{*}\right)\left(1-{T^{*}}^{1.096}\right)$$

### 3.2. Modified Johnson–Cook Model (LMJCM)

Lin et al. [60] introduced a well-known modification for the OJCM to predict the flow behavior of high-strength alloy steel at elevated temperatures. The interaction between the strain rate and temperature is considered in this modification. The LMJCM is introduced as:

(9) $$\sigma =\left(A+{B_{1}\varepsilon +B_{2}\varepsilon }^{2}\right)\left(1+C_{1}\ln{\dot{\varepsilon }}^{*}\right)\exp\left[\left(\lambda_{1}+\lambda_{2}\ln{\dot{\varepsilon }}^{*}\right)\left(T-T_{r}\right)\right]$$

where σ is the flow stress, ε is the plastic strain, and $A,\;B_{1},\;B_{2},\;C_{1},\;\lambda_{1},$ and $\lambda_{2}$ are material constants. ${\dot{\varepsilon }}^{*}=\dot{\varepsilon }/{\dot{\varepsilon }}_{\circ }$ is a dimensionless strain rate where $\dot{\varepsilon }$ and ${\dot{\varepsilon }}_{\circ }$ are the strain rate and a chosen reference strain rate, respectively. Additionally, T and $T_{r}$ are the tested temperatures and a chosen reference temperature, respectively.

At a reference temperature of 400 °C and a reference strain rate of 0.001 s^−1^, Equation (9) decreases to:

(10) $$\sigma =A+{B_{1}\varepsilon +B_{2}\varepsilon }^{2}$$

**Figure 2:**
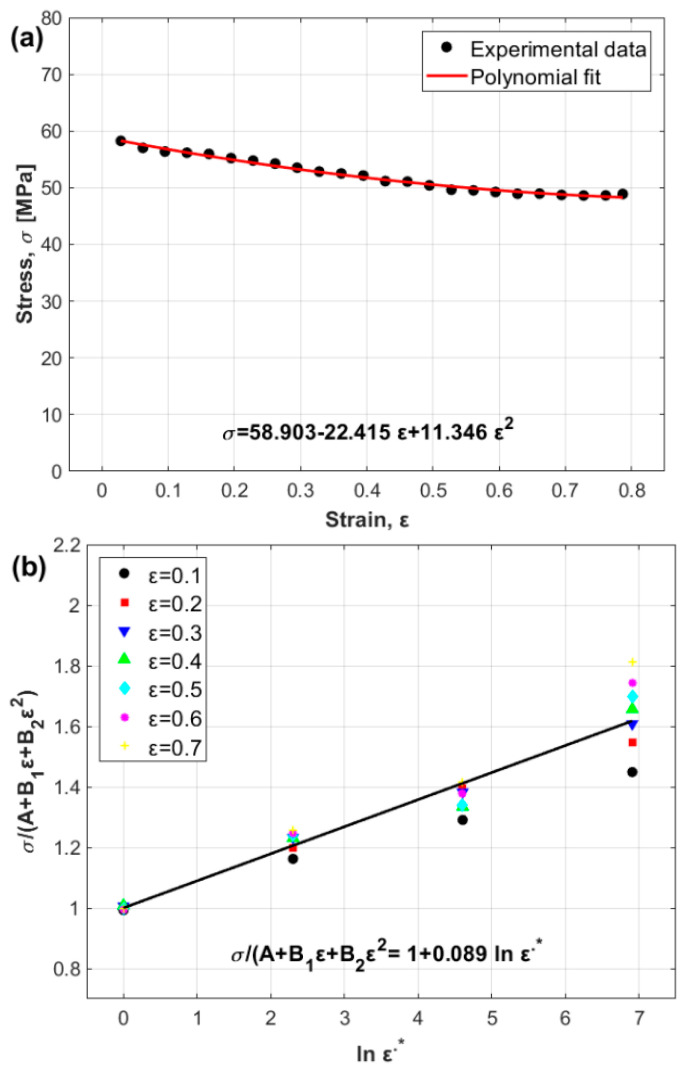
(**a**) Stress versus strain from reference data and (**b**) $\sigma /\left(A+{B_{1}\varepsilon +B_{2}\varepsilon }^{2}\right)$ versus $\ln{\dot{\varepsilon }}^{*}$.

The constants A, $B_{1}$, and $B_{2}$ are determined to be 58.903 MPa, −22.415 MPa, and 11.346 MPa, respectively, by fitting with a second-order polynomial, as shown in Figure 2a.

At the reference temperature of 400 °C, Equation (9) reduces to:

(11) $$\sigma =\left(A+{B_{1}\varepsilon +B_{2}\varepsilon }^{2}\right)\left(1+C_{1}\ln{\dot{\varepsilon }}^{*}\right)$$

After some rearrangements are performed, Equation (11) can be written as:

(12) $$\frac{\sigma }{A+{B_{1}\varepsilon +B_{2}\varepsilon }^{2}}=1+C_{1}\ln{\dot{\varepsilon }}^{*}$$

The constant $C_{1}$ is the slope and is determined to be 0.089 by plotting $\sigma /\left(A+{B_{1}\varepsilon +B_{2}\varepsilon }^{2}\right)$ versus $\ln{\dot{\varepsilon }}^{*}$ at different strain rates and strains from 0.1 to 0.7 with an increment of 0.1, as shown in Figure 2b.

At different temperatures and strain rates, after some rearrangements are performed, Equation (9) can be expressed as:

(13) $$\frac{\sigma }{\left(A+{B_{1}\varepsilon +B_{2}\varepsilon }^{2}\right)\left(1+C_{1}\ln{\dot{\varepsilon }}^{*}\right)}=e^{\lambda \left(T-T_{r}\right)}$$

Taking the logarithm of both sides, Equation (13) can be introduced as:

(14) $$\ln\left[\frac{\sigma }{\left(A+{B_{1}\varepsilon +B_{2}\varepsilon }^{2}\right)\left(1+C_{1}\ln{\dot{\varepsilon }}^{*}\right)}\right]=\lambda \left(T-T_{r}\right)$$

**Figure 3:**
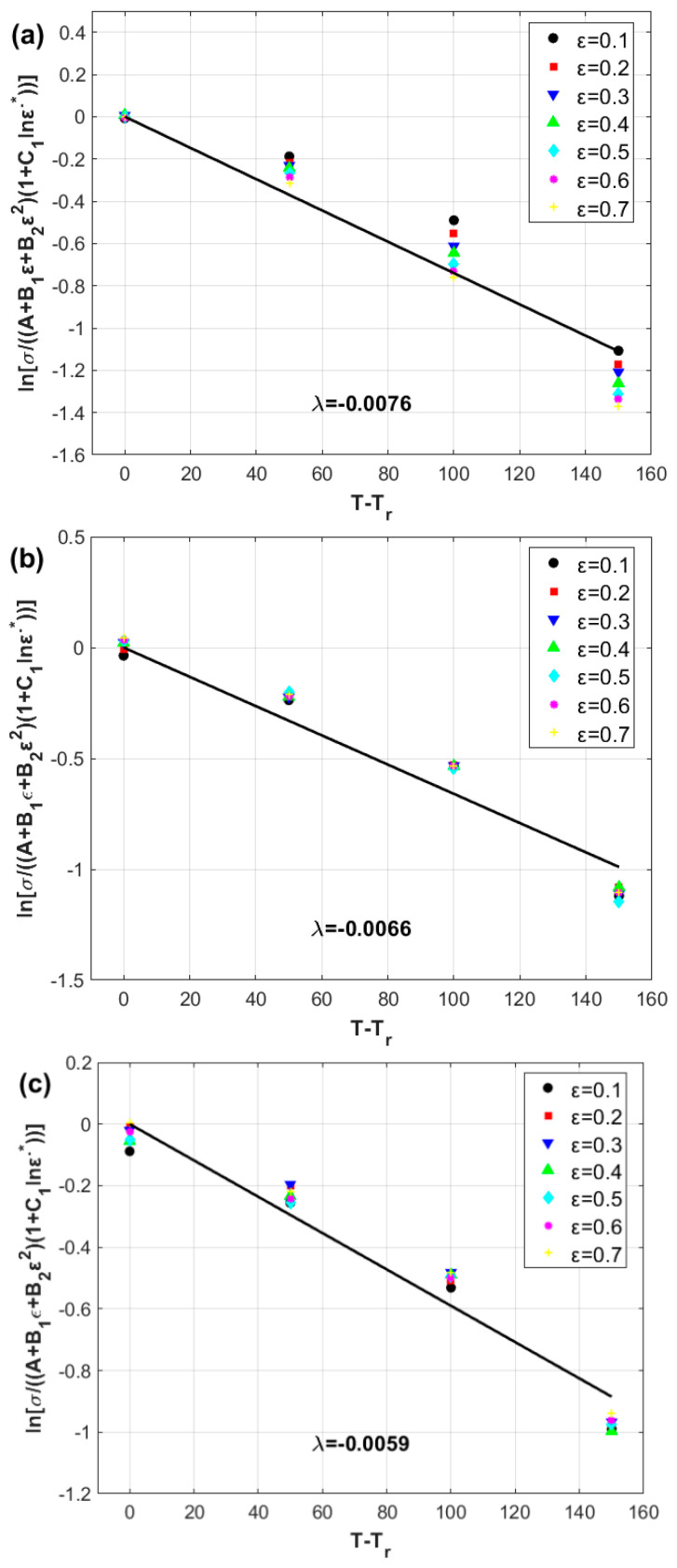

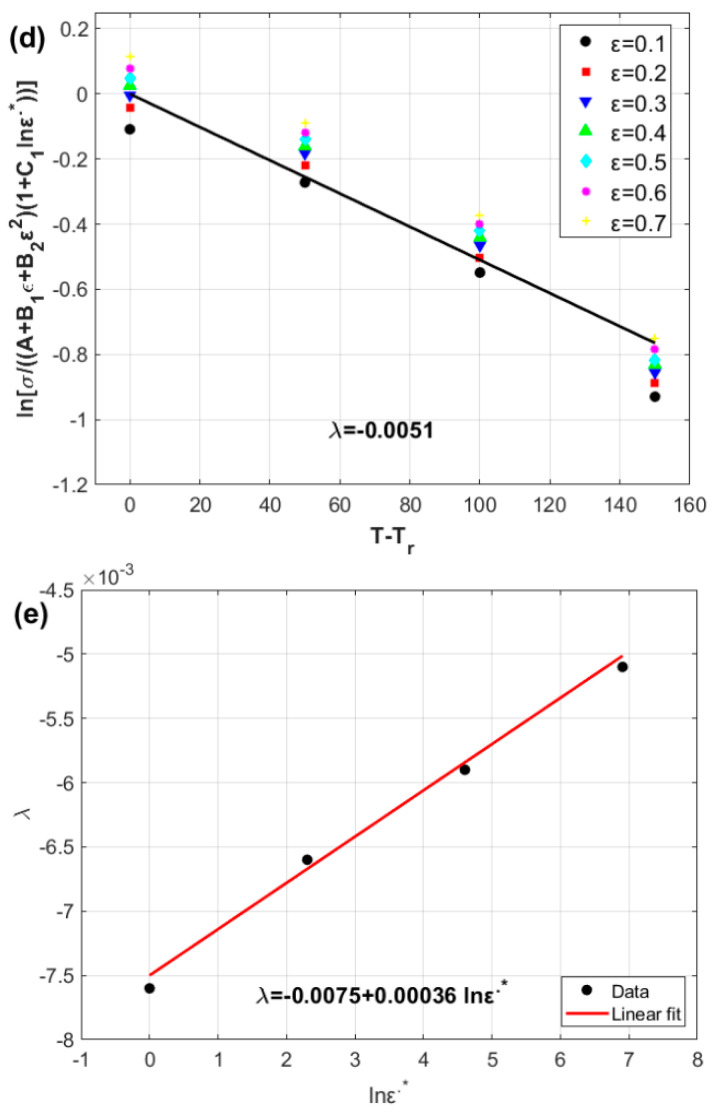
Determination of the modified JC constants $\lambda ,\;\lambda_{1},$ and $\lambda_{2}$ (**a**) λ at 0.001 s^−1^, (**b**) λ at 0.01 s^−1^, (**c**) λ at 0.1 s^−1^, (**d**) λ at 1 s^−1^, and (**e**) $\lambda_{1}$ and $\lambda_{2}$.

By plotting $\ln\left[\sigma /\left(\left(A+{B_{1}\varepsilon +B_{2}\varepsilon }^{2}\right)\left(1+C_{1}\ln{\dot{\varepsilon }}^{*}\right)\right)\right]$ versus $\left(T-T_{r}\right)$, four different values for λ accompanied by the four values of temperature (slope of the equation, Figure 3a–d) are determined. The values are −0.0076, −0.0066, −0.0059, and −0.0051 for temperatures of 400 °C, 450 °C, 500 °C, and 550 °C, respectively. By plotting the obtained values of λ versus $\ln{\dot{\varepsilon }}^{*}$ via linear regression, the values of $\lambda_{1}$ and $\lambda_{2}$ can be determined as −0.0075 and 0.00036, respectively, in which $\lambda_{1}$ is the intercept and $\lambda_{2}$ is the slope (Figure 3e).

Thus, the LMJCM can be introduced as:

(15) $$\sigma =\left(58.903-{22.415\;\varepsilon +11.346\;\varepsilon }^{2}\right)\left(1+0.089\ln{\dot{\varepsilon }}^{*}\right)\exp\left[\left(-0.0075+\;0.00036\ln{\dot{\varepsilon }}^{*}\right)\left(T-T_{r}\right)\right]$$

### 3.3. Proposed Modification of the Johnson–Cook (PMJCM)

Based on OJMM and LMJCM, which are represented in Equations (1) and (9), respectively, a general proposed modification of the OJCM can be written as:

(16) $$\sigma =\left(A+{B_{1}\varepsilon +B_{2}\varepsilon }^{2}+{B_{3}\varepsilon }^{3}\right)\left(1+C\left(\varepsilon ,\ln{\dot{\varepsilon }}^{*}\right)\ln{\dot{\varepsilon }}^{*}\right)\exp\left[D\left(\varepsilon ,\ln{\dot{\varepsilon }}^{*},T^{s}\right){\;T}^{s}\right]$$

where ${\;T}^{s}=\left({T-T}_{r}\right)/T_{r}$, σ, ε, T, and $T_{r}$ are defined as in the previous subsections, and $A,\;B_{1},\;B_{2}$, and $B_{3}$ are material constants.

At a reference temperature of 400 °C and a reference strain rate of 0.001 s^−1^, Equation (16) decreases to:

(17) $$\sigma =A+{B_{1}\varepsilon +B_{2}\varepsilon }^{2}+{B_{3}\varepsilon }^{3}$$

**Figure 4:**
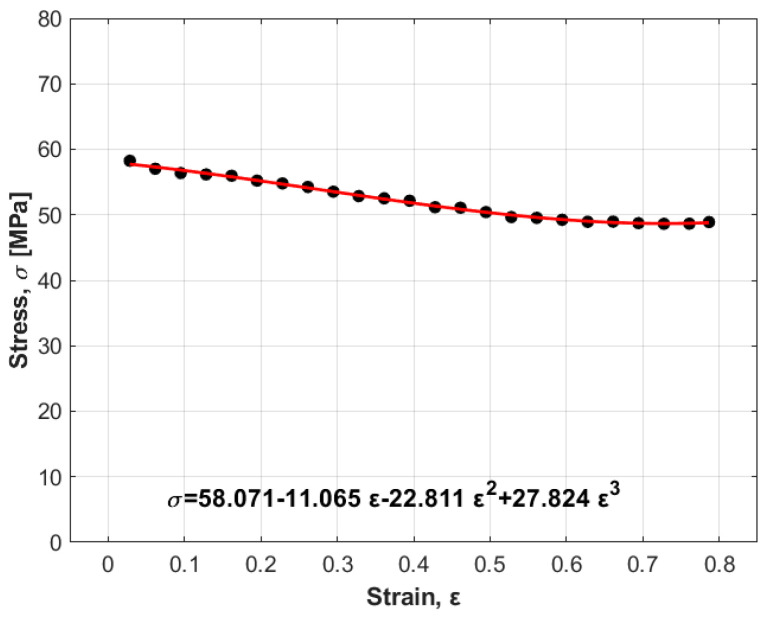
Stress vs. strain at the reference data (circles are for experimental data, and solid line is for fitting).

The constants $A,\;B_{1},\;B_{2}$, and $B_{3}$ are determined to be 58.071 MPa, −11.065 MPa, −22.811 MPa, and 27.824 MPa, respectively, via quadratic fitting, as shown in Figure 4.

At the reference temperature of 400 °C, Equation (16) reduces to:

(18) $$\sigma =\left(A+{B_{1}\varepsilon +B_{2}\varepsilon }^{2}+{B_{3}\varepsilon }^{3}\right)\left(1+C\left(\varepsilon ,\ln{\dot{\varepsilon }}^{*}\right)\ln{\dot{\varepsilon }}^{*}\right)$$

After some rearrangements are performed, the parameter $C\left(\varepsilon ,\ln{\dot{\varepsilon }}^{*}\right)$ in Equation (18) can be written as:

(19) $$C\left(\varepsilon ,\ln{\dot{\varepsilon }}^{*}\right)=\left(\frac{\sigma }{A+{B_{1}\varepsilon +B_{2}\varepsilon }^{2}+{B_{3}\varepsilon }^{3}}-1\right)/\ln{\dot{\varepsilon }}^{*}$$

Considering a reference temperature of 400 °C and strain rates of 0.001 s^−1^, 0.01 s^−1^, and 1 s^−1^, different values of the parameter $C\left(\varepsilon \right)$ at different strains, ε, can be computed, as shown in Table 1. Considering the strain rate effect, different values of the parameter $C\left(\ln{\dot{\varepsilon }}^{*}\right)$ at different temperatures can be computed, as shown in Table 2.

**Figure 5:**
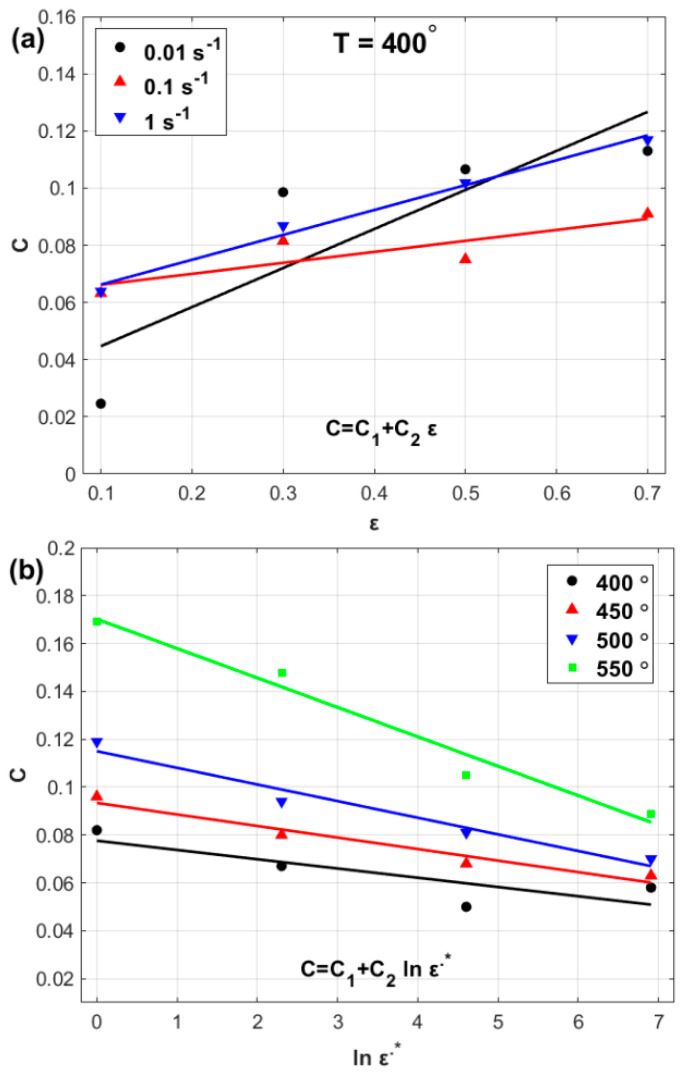
(**a**) *C* versus ε, and (**b**) *C* versus $\ln{\dot{\varepsilon }}^{*}$.

By plotting the obtained values of *C*(*ε*) (Table 1) against *ε* and the obtained values of $C\left(\ln{\dot{\varepsilon }}^{*}\right)$ (Table 2) against $\ln{\dot{\varepsilon }}^{*}$, a linear relationship between parameter C and both *ε* and $\ln{\dot{\varepsilon }}^{*}$ can be observed, as shown in Figure 5. Therefore, $C\left(\varepsilon ,\ln{\dot{\varepsilon }}^{*}\right)$ can be expressed as:

(20) $$C\left(\varepsilon ,\ln{\dot{\varepsilon }}^{*}\right)=C_{1}+C_{2}\varepsilon +C_{3}\;\ln{\dot{\varepsilon }}^{*}+C_{4}\;\varepsilon \ln{\dot{\varepsilon }}^{*}$$

Considering all the temperature values and strain rates and at different strain values, the parameter $D\left(\varepsilon ,\ln{\dot{\varepsilon }}^{*},T^{s}\right)$ can be expressed via Equation (16) as:

(21) $$D\left(\varepsilon ,\ln{\dot{\varepsilon }}^{*},T^{s}\right)=\left[\ln\frac{\sigma }{\left(A+{B_{1}\varepsilon +B_{2}\varepsilon }^{2}+{B_{3}\varepsilon }^{3}\right)\left(1+C\left(\varepsilon ,\ln{\dot{\varepsilon }}^{*}\right)\ln{\dot{\varepsilon }}^{*}\right)}\right]/T^{s}$$

At a selected temperature of 450 °C and different strain rate values, $D\left(\varepsilon \right)$ can be computed as shown in Table 3. Thus, at a selected temperature of 450 °C, different strain values $D\left(\ln{\dot{\varepsilon }}^{*}\right)$ can be computed, as shown in Table 4. Furthermore, at a selected strain rate of 0.01 s^−1^ and different strain values, $D\left(T^{s}\right)$ can be computed as shown in Table 5.

**Figure 6:**
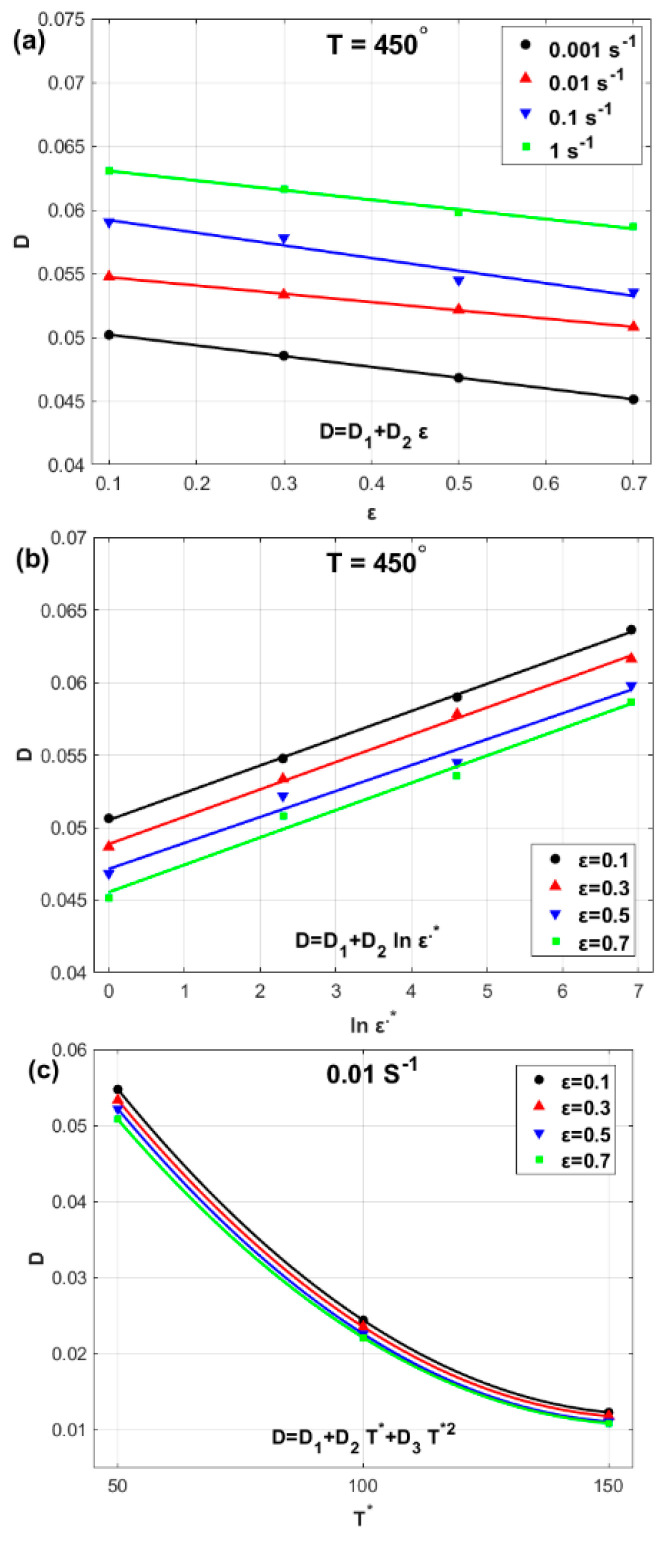
(**a**) *D* versus ε, (**b**) *D* versus $\ln{\dot{\varepsilon }}^{*}$, and (**c**) *D* versus $T^{*}$.

The obtained values of parameter $D\left(\varepsilon ,\ln{\dot{\varepsilon }}^{*},T^{s}\right)$ which are presented in Table 3, Table 4 and Table 5, can be plotted against $\varepsilon ,\ln{\dot{\varepsilon }}^{*}$, and, $T^{s}$ as shown in Figure 6. The figure shows that linear relationships can be expressed between D and both ε (Figure 6a) and $\ln{\dot{\varepsilon }}^{*}$ (Figure 6b), whereas quadratic fitting can be achieved between D and $T^{s}$ (Figure 6c). Accordingly, the parameter $D\left(\varepsilon ,\ln{\dot{\varepsilon }}^{*},T^{s}\right)$ can be introduced as:

(22) $$D\left(\varepsilon ,\ln{\dot{\varepsilon }}^{*},T^{s}\right)=D_{1}+D_{2}\varepsilon +D_{3}\ln{\dot{\varepsilon }}^{*}+D_{4}{T^{s}}^{2}+D_{5}\varepsilon \;\ln{\dot{\varepsilon }}^{*}\;T^{s}$$

Finally, the PMJCM can be expressed as:

(23) $$\sigma =\left(A+{B_{1}\varepsilon +B_{2}\varepsilon }^{2}+{B_{3}\varepsilon }^{3}\right)\;\left(1+\left(C_{1}+C_{2}\;\varepsilon +C_{3}\;\ln{\dot{\varepsilon }}^{*}+C_{4}\;\varepsilon \ln{\dot{\varepsilon }}^{*}\right)\ln{\dot{\varepsilon }}^{*}\right)\exp\left[\left(D_{1}+D_{2}\;\varepsilon +D_{3}\ln{\dot{\varepsilon }}^{*}+D_{4}\;{T^{s}}^{2}+D_{5}\;\varepsilon \;\ln{\dot{\varepsilon }}^{*}\;T^{s}\right)\;T^{s}\right]$$

A Kalman filter is an inverse mathematical method implemented to determine the PMJCM constants $C_{1},\;C_{2},\;C_{3},\;C_{4},\;D_{1},\;D_{2},\;D_{3},\;D_{4},\;and\;D_{5}$. The Kalman filter uses the nonlinear least squares method to minimize the mean square errors between the predicted and measured data. The Kalman filter equation is given by [74]:

(24) $$\beta_{t+1}=\beta_{t}+K\left(\sigma_{exp}-\sigma \left(\beta_{t}\right)\right)$$

where $\beta_{t+1}$ is an n×1 vector containing model parameters at iteration t and where n is the number of parameters. The measured stresses are included in an N×1 vector named $\sigma_{exp}$, where N is the number of measurements. $\sigma \left(\beta_{t}\right)$ contains the predicted stresses at $\beta_{t}$. The parameters $K_{t}$ introduce a Kalman gain, which is an n×N matrix and is always updated as:

(25) $$K_{t}=P_{t}H_{t}^{T}{\left(H_{t}P_{t}H_{t}^{T}+R\right)}^{-1}$$

where $P_{t}$ represents an n×n matrix, which considers the covariance error of the seeking parameters and is always updated as:

(26) $$P_{t+1}=\left(I-K_{t}H_{t}\right)P_{t}+Q$$

where R and Q introduce covariance errors for both the measurements and the parameters. The matrix H introduces a Jacobian matrix with N×n, which represents the derivatives of the predicted stresses with respect to the predicted parameters [74].

The constants $A,\;B_{1},\;B_{2}$, and $B_{3}$ are determined to be 58.071 MPa, −11.065 MPa, −22.811 MPa, and 27.824 MPa, respectively, via quadratic fitting, as described in Section 3.3 (Figure 4).

At a reference temperature of 450 °C, Equation (23) decreases to:

(27) $$\sigma =\left(A+{B_{1}\varepsilon +B_{2}\varepsilon }^{2}+{B_{3}\varepsilon }^{3}\right)\left(1+\left(C_{1}+C_{2}\;\varepsilon +C_{3}\;\ln{\dot{\varepsilon }}^{*}+C_{4}\;\varepsilon \ln{\dot{\varepsilon }}^{*}\right)\ln{\dot{\varepsilon }}^{*}\right)$$

Using the Kalman filter, the parameter vector β is given by ${\left(C_{1}C_{2}C_{3}C_{4}\right)}^{T}$, which contains strain rate parameters. The Kalman filter is implemented again to determine the softening parameters. Using Equation (23) at different temperatures and strain rates, β is given by ${\left(D_{1}D_{2}D_{3}D_{4}D_{5}\right)}^{T}$. The initial values for the four strain rate parameters and the five softening parameters are suggested based on an analysis of the mean square error between the measured values and those predicted via many initial parameters.

**Figure 7:**
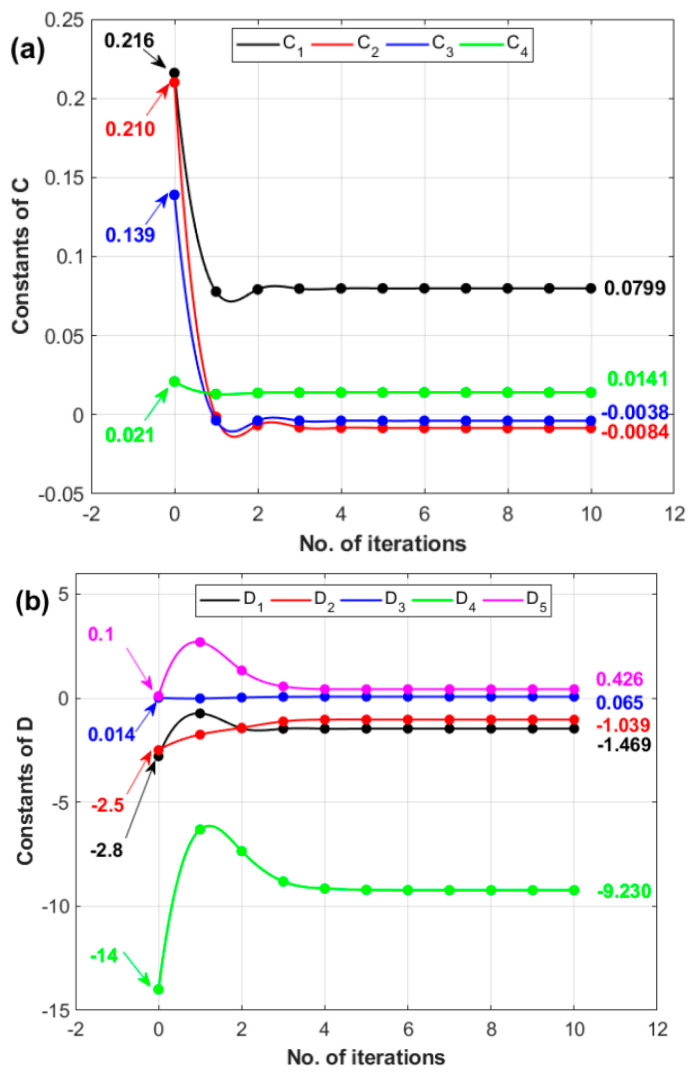
Number of iterations versus (**a**) constants of *C* and (**b**) constants of *D*.

Initial values for C constants, i.e., $C_{1},C_{2},C_{3}$, and $C_{4}$, with 0.216, 0.216, 0.139, and 0.021, and for D constants, i.e., $D_{1},D_{2},D_{3},D_{4}$, and $D_{5}$, with −2.8, −2.5, 0.014, −14, and 0.1, are tracked against the number of iterations and shown in Figure 7a,b, respectively. The initial values are marked with arrows in the figure. The figure shows that the convergence of the constants is almost achieved after five iterations. The constants $C_{1},C_{2},C_{3}$, and $C_{4}$ are determined to be 0.0799, −0.0084, −0.0038, and 0.0141 (Figure 7a), and the constants $D_{1},D_{2},D_{3},D_{4}$, and $D_{5}$ are determined to be −1.469, −1.039, 0.065, −9.230, and 0.426 (Figure 7b).

Hence, the final PMJCM can be expressed as:

(28) $$\begin{array}{cc} \sigma = & \left(58.07-11.06\;{\varepsilon -22.81\;\varepsilon }^{2}+{27.82\;\varepsilon }^{3}\right)\\ & \left(1+\left(0.0799-0.0084\;\varepsilon -0.0038\;\ln{\dot{\varepsilon }}^{*}\right.\right.\\ & \left.\left.+0.0141\;\varepsilon \ln{\dot{\varepsilon }}^{*}\right)\ln{\dot{\varepsilon }}^{*}\right)\exp\left[\left(-1.469-1.039\;\varepsilon +0.065\ln{\dot{\varepsilon }}^{*}\right.\right.\\ & \left.\left.-9.23\;{T^{s}}^{2}+0.426\;\varepsilon \;\ln{\dot{\varepsilon }}^{*}\;T^{s}\right){\;T}^{s}\right] \end{array}$$

## 4. Results and Discussion

### 4.1. Predicted Stresses Compared with Experimental Stresses

In this subsection, a comparison between the predicted stresses obtained by the OJMM, LMJCM, and PMJCM and the experimental stresses is presented and addressed.

**Figure 8:**
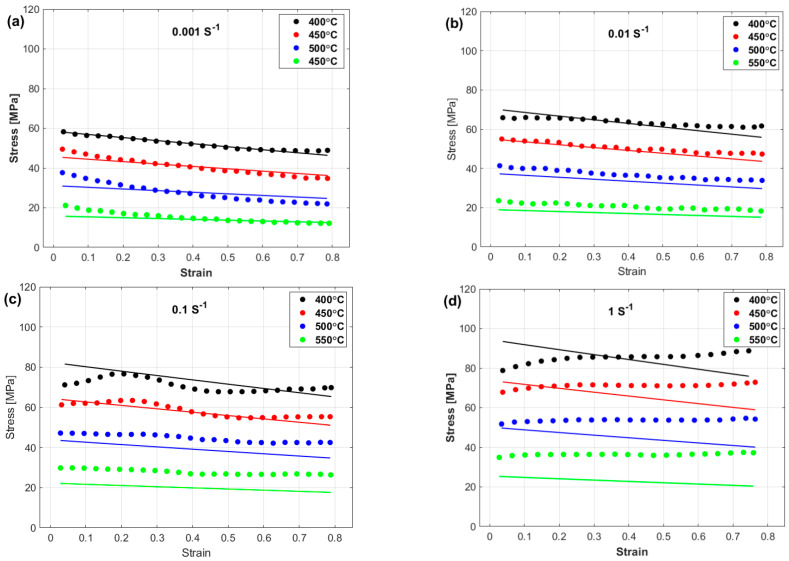
Experimental stresses (markers) versus predicted stresses obtained by the OJCM (lines) at (**a**) 0.001 s^−1^, (**b**) 0.01 s^−1^, (**c**) 0.1 s^−1^, and (**d**) 1 s^−1^.

The experimental stresses and predicted stresses obtained via the OJCM are compared and shown in Figure 8. The figure shows that the OJCM may yield good predictions only at the reference strain rate (Figure 8a), whereas it fails to accurately predict the flow behavior at the left strain rate and temperature values (Figure 8b–d). A possible reason is that the strain, strain rate, and temperature are not correlated with each other in the OJCM.

**Figure 9:**
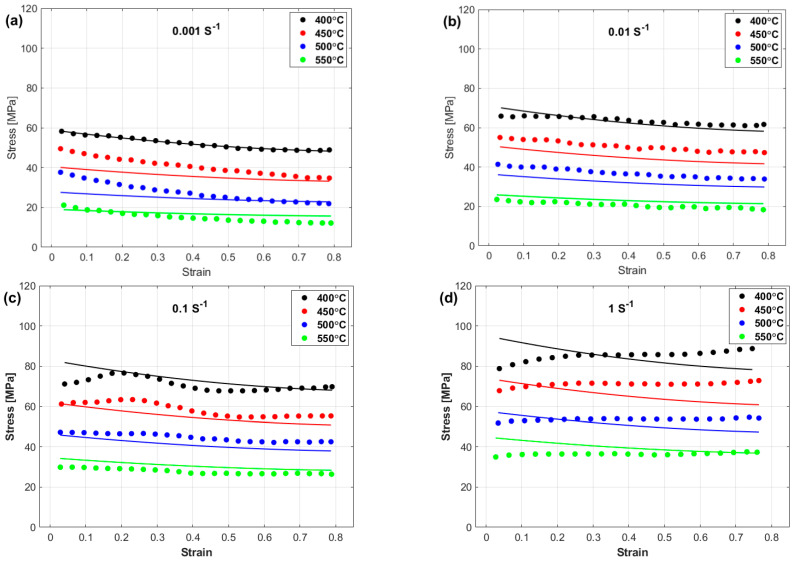
Experimental stresses (markers) versus predicted stresses obtained by the LMJCM (lines) at (**a**) 0.001 s^−1^, (**b**) 0.01 s^−1^, (**c**) 0.1 s^−1^, and (**d**) 1 s^−1^.

Figure 9 shows a comparison between the experimental stresses and the stresses predicted by the LMJCM. As shown, the LMJCM did not capture the flow behavior precisely, which might be related to the complex behavior of the tested alloy during hot deformation.

**Figure 10:**
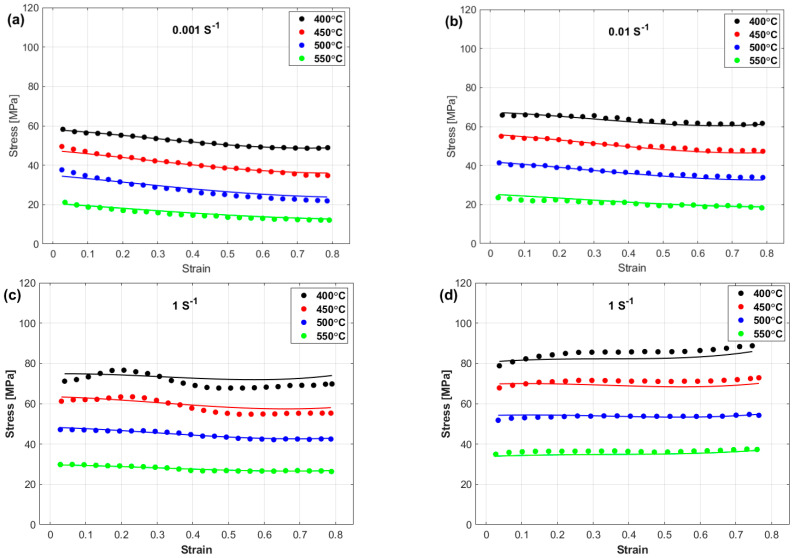
Experimental stresses (markers) versus predicted stresses obtained by the PMJCM (lines) at (**a**) 0.001 s^−1^, (**b**) 0.01 s^−1^, (**c**) 0.1 s^−1^, and (**d**) 1 s^−1^.

Finally, a comparison between the experimental stresses and predicted stresses obtained via the PMJCM is shown in Figure 10. The figure shows that the PMJCM can precisely predict the flow behavior of the tested alloy with all the combinations of temperatures and strain rates. The PMJCM considers the interaction between strain, strain rate, and temperature, which might be a reason for the complex nonlinear behavior of the tested alloy during hot deformation.

### 4.2. Assessment and Evaluation of the Models

The predictabilities of the OJCM, LMJCM, and PMJCM models are evaluated and assessed via the statistical parameters R, AARE, and RMSE, which are computed as [52,75,76]:

(29) $$R=\frac{\sum_{i}^{N}\left(\sigma_{e}-\bar{\sigma_{e}}\right)\left(\sigma_{P}-\bar{\sigma_{P}}\right)}{\sqrt{\sum_{i}^{N}{\left(\sigma_{e}-\bar{\sigma_{e}}\right)}^{2}\sum_{i}^{N}{\left(\sigma_{P}-\bar{\sigma_{P}}\right)}^{2}}}$$

(30) $$AARE=\frac{1}{N}\sum_{i}^{N}\left|\frac{\sigma_{e}-\sigma_{P}}{\sigma_{e}}\right|\times 100$$

(31) $$RMSE=\frac{1}{N}\sqrt{\sum_{i}^{N}{\left(\sigma_{e}-\sigma_{P}\right)}^{2}}$$

where $\sigma_{e}$ and $\bar{\sigma_{e}}$ represent the experimental stresses and their mean values, respectively, while $\sigma_{P}$ and $\bar{\sigma_{P}}$ represent the predicted stresses and their mean values, respectively, and N represents the total number of observations.

**Figure 11:**
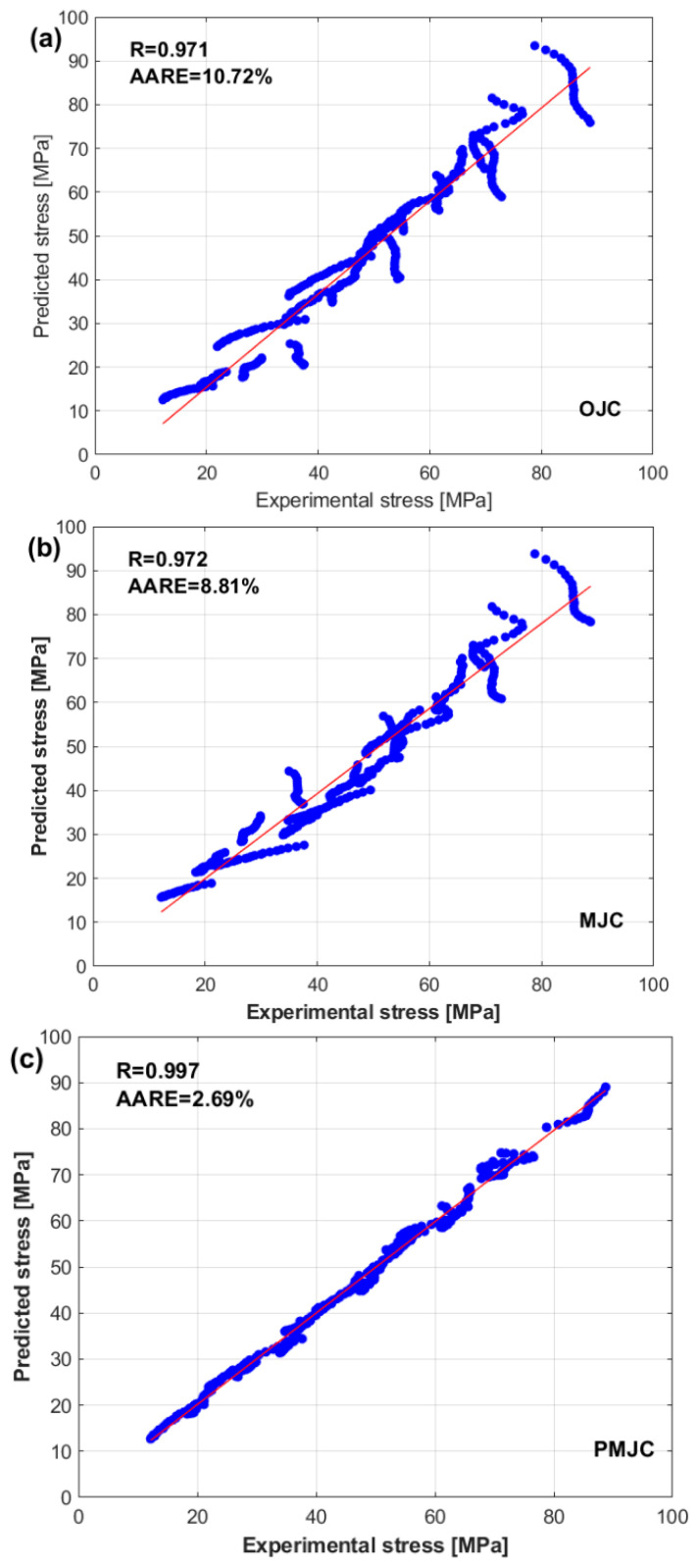
Correlation of the experimental stresses and predicted stresses (dots) obtained by the (**a**) OJCM model, (**b**) LMJCM, (**c**) and PMJCM. The solid line represents the regression line.

A correlation between the experimental stresses and the stresses predicted via the OJCM, LMJCM, and PMJCM via Equation (29) is shown in Figure 11. The figure shows that the PMJCM yields the best R value of 0.997, and both the LMJCM and OJCM yield values close to each other, with values of 0.972 and 0.971, respectively.

**Figure 12:**
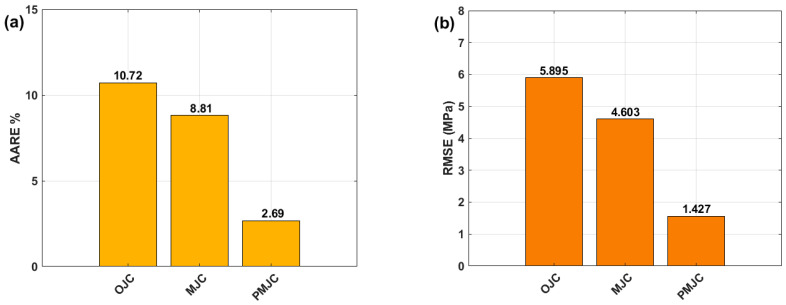
Bar charts for (**a**) AARE (%) and (**b**) RMSE (MPa), obtained by the OJCM, LMJCM, and PMJCM models.

The AARE values obtained by the OJCM, LMJCM, and PMJCM are plotted in Figure 12a. The PMJCM gives the best and lowest AARE, with a value of 2.69%, whereas both the LMJCM and OJCM yield values of 8.81% and 10.72%, respectively. Figure 12 shows that the lowest RMSE value is also obtained by the PMJCM, with a value of 1.427 MPa, and the LMJCM and OJCM are in second and third place, with values of 4.603 MPa and 5.895 MPa, respectively.

## 5. Conclusions

The key conclusions from this investigation can be summarized as follows:

- The OJCM of AA6082 provides good predictions only at the reference strain rate, whereas it fails to accurately predict the flow behavior at the remaining $\dot{\varepsilon }$ and T. This is attributed to the fact that $\dot{\varepsilon }$, ε, and T are not correlated with each other in this model. The LMJCM of AA6082 failed to capture the flow behavior precisely, which might be related to the complex behavior of the AA6082 alloy during hot deformation.
- The PMJCM of AA6082 is proposed in this study as follows: $\sigma =\left(A+{B_{1}\varepsilon +B_{2}\varepsilon }^{2}+{B_{3}\varepsilon }^{3}\right)\left(1+\left(C_{1}+C_{2}\;\varepsilon +C_{3}\;\ln{\dot{\varepsilon }}^{*}+C_{4}\;\varepsilon \ln{\dot{\varepsilon }}^{*}\right)\ln{\dot{\varepsilon }}^{*}\right)\exp\left[\left(D_{1}+D_{2}\;\varepsilon {+D}_{3}\ln{\dot{\varepsilon }}^{*}+\;D_{4}\;{T^{s}}^{2}{+D}_{5}\;\varepsilon \;\ln{\dot{\varepsilon }}^{*}\;T^{s}\right)\;T^{s}\right]$. The Kalman filter was implemented to determine the PMJCM constants $C_{1}$, $C_{2}$, $C_{3}$, $C_{4}$, $D_{1}$, $D_{2}$, $D_{3}$, $D_{4}$, and $D_{5}$. The constants $C_{1},{\;C}_{2},{\;C}_{3}$ and $C_{4}$ are determined to be 0.0799, −0.0084, −0.0038, and 0.0141, and the constants $D_{1},D_{2},D_{3},\;D_{4}$, and $D_{5}$ are determined to be −1.469, −1.039, 0.065, −9.230, and 0.426. Thus, the final PMJCM can be expressed as $\sigma =\left(58.07-11.06\;{\varepsilon -22.81\;\varepsilon }^{2}+{27.82\;\varepsilon }^{3}\right)\left(1+\left(0.0799-0.0084\;\varepsilon -0.0038\;\ln{\dot{\varepsilon }}^{*}+0.0141\;\varepsilon \ln{\dot{\varepsilon }}^{*}\right)\ln{\dot{\varepsilon }}^{*}\right)\exp\left[\left(-1.469-1.039\;\varepsilon +0.065\ln{\dot{\varepsilon }}^{*}-9.23\;{T^{s}}^{2}+0.426\;\varepsilon \;\ln{\dot{\varepsilon }}^{*}\;T^{s}\right){\;T}^{s}\right]$.
- The stresses predicted by the PMJCM closely match the experimental stresses because of the interaction between $\dot{\varepsilon }$, ε, and T, which might be a reason for the complex nonlinear behavior of the AA6082 alloy during hot deformation. In addition, the PMJCM gives the best R value of 0.997, and both the LMJCM and OJCM give values close to 0.972 and 0.971, respectively. In addition, the PMJCM gives the best and lowest AARE, with a value of 2.69%, whereas both the LMJCM and OJCM come next with values of 8.81% and 10.72%, respectively. In terms of the RMSE, the PMJCM gives the lowest value of 1.427 MPa, and the MJC and OJC are in second and third place, with values of 4.603 MPa and 5.895 MPa, respectively. This indicates that PMJCM gives a significantly better fit than the LMJCM and OJCM.

## Figures and Tables

**Table 1:** Values of *C*(*ε*) at reference T and different ε and $\dot{\varepsilon }$ values.

	Strain Rate s^−1^		
Strain	0.01	0.1	1
0.1	0.02460	0.06326	0.06385
0.3	0.09856	0.08146	0.08681
0.5	0.10657	0.07504	0.10181
0.7	0.11299	0.09101	0.11687

**Table 2:** Values of $C(\ln{\dot{\varepsilon }}^{*})$ at different T and $\dot{\varepsilon }$ values.

	Temperature °C			
$\ln{\dot{\varepsilon }}^{*}$	400	450	500	550
0	0.082	0.096	0.119	0.169
2.30259	0.067	0.08	0.094	0.148
4.60517	0.050	0.068	0.081	0.105
6.90776	0.058	0.063	0.070	0.089

**Table 3:** Values of D(ε) at a selected temperature of 450 °C and different strain values.

	Strain Rate s^−1^			
Strain	0.001	0.01	0.1	1
0.1	0.05019	0.05475	0.05905	0.06308
0.3	0.04857	0.05334	0.05780	0.06162
0.5	0.04683	0.05217	0.05448	0.05978
0.7	0.04514	0.05081	0.05355	0.05866

**Table 4:** Values of $D\left(\ln{\dot{\varepsilon }}^{*}\right)$ at a selected temperature of 450 °C and different strain rate values.

	Strain			
$\ln{\dot{\varepsilon }}^{*}$	0.1	0.3	0.5	0.7
0	0.05062	0.04866	0.04683	0.04514
2.30259	0.05475	0.05334	0.05217	0.05081
4.60517	0.05899	0.05780	0.05448	0.05355
6.90776	0.06362	0.06162	0.05978	0.05866

**Table 5:** Values of $D(T^{s})$ at a selected strain rate of 0.01 s^−1^ and different temperatures.

	Strain			
Temperature °C	0.1	0.3	0.5	0.7
450	0.05475	0.05334	0.05217	0.05081
500	0.02441	0.02354	0.02262	0.02214
550	0.01232	0.01183	0.01111	0.01090

## Data Availability

Data will be available upon request through the corresponding author.

## References

[1] Abd El-Aty A., Xu Y., Guo X., Zhang S., Ma Y., Chen D. (2018). Strengthening mechanisms, deformation behavior, and anisotropic mechanical properties of Al-Li alloys: A review. J. Adv. Res..

[2] Alami A.H., Olabi A.G., Alashkar A., Alasad S., Aljaghoub H., Rezk H., Abdelkareem M.A. (2023). Additive manufacturing in the aerospace and automotive industries: Recent trends and role in achieving sustainable development goals. Ain Shams Eng. J..

[3] Jarfors A.E.W., Du A., Yu G., Zheng J., Wang K. (2020). On the sustainable choice of alloying elements for strength of aluminum-based alloys. Sustainability.

[4] Aljaghoub H., Abumadi F., AlMallahi M.N., Obaideen K., Alami A.H. (2022). Solar PV cleaning techniques contribute to sustainable development goals (SDGs) using multi-criteria decision-making (MCDM): Assessment and review. Int. J. Thermofluids.

[5] Sun Y. (2023). The use of aluminum alloys in structures: Review and outlook. Structures.

[6] Blanco D., Rubio E.M., Lorente-Pedreille R.M., Sáenz-Nuño M.A. (2022). Sustainable processes in aluminium, magnesium, and titanium alloys applied to the transport sector: A review. Metals.

[7] Cann J.L., De Luca A., Dunand D.C., Dye D., Miracle D.B., Oh H.S., Olivetti E.A., Pollock T.M., Poole W.J., Yang R. (2021). Sustainability through alloy design: Challenges and opportunities. Prog. Mater. Sci..

[8] Pramanik A., Basak A.K., Prakash C., Shankar S., Chattopadhyaya S. (2022). Sustainability in drilling of aluminum alloy. Clean. Mater..

[9] Emadi P., Andilab B., Ravindran C. (2022). Engineering lightweight aluminum and magnesium alloys for a sustainable future. J. Indian Inst. Sci..

[10] Hou Y., Myung D., Park J.K., Min J., Lee H.R., El-Aty A.A., Lee M.G. (2023). A review of characterization and modelling approaches for sheet metal forming of lightweight metallic materials. Materials.

[11] Heidarzadeh A., Khorshidi M., Mohammadzadeh R., Khajeh R., Mofarrehi M., Javidani M., Chen X.G. (2023). Multipass friction stir processing of laser-powder bed fusion AlSi10Mg: Microstructure and mechanical properties. Materials.

[12] Xia L., Zhang S.H., Xu Y., Chen S., Abd El-Aty A., Pokrovsky A.I., Bakinovskaya A.A. (2021). Study of the ductility enhancement of 5A90 Al–Mg–Li alloy sheets with stress relaxation. Philos. Mag..

[13] Cao L., Liao B., Wu X., Li C., Huang G., Cheng N. (2020). Hot deformation behavior and microstructure characterization of an Al-Cu-Li-Mg-Ag alloy. Crystals.

[14] Zheng K., Politis D., Wang L., Lin J. (2018). A review on forming techniques for manufacturing lightweight complex-shaped aluminium panel components. Int. J. Lightweight Mater. Manuf..

[15] Lin Y., Chen X. (2011). A critical review of experimental results and constitutive descriptions for metals and alloys in hot working. Mater. Des..

[16] Hu M., Sun Y., He J., Li C., Li H., Yu L., Liu Y. (2021). Hot deformation behaviour and microstructure evolution of Al-3%Mg_2_Si alloy. Mater. Charact..

[17] Liang H., Nan Y., Ning Y., Li H., Zhang L., Shi Z., Guo H. (2013). Correlation between strain-rate sensitivity and dynamic softening behavior during hot processing. J. Alloys Compd..

[18] Lin Y.C., Li L.T., Xia Y.C., Jiang Y.Q. (2013). Hot deformation and processing map of a typical Al-Zn-Mg-Cu alloy. J. Alloys Compd..

[19] Liang Z., Zhang Q., Niu L., Luo W. (2019). Hot deformation behavior and processing maps of as-cast hypoeutectic Al-Si-Mg alloy. J. Mater. Eng. Perform..

[20] Huang Y., Liu L., Xiao Z., Wang S. (2019). Hot deformation behavior of 6063 aluminum alloy studied using processing maps and microstructural analysis. Phys. Met. Metallogr..

[21] Abedrabbo N., Pourboghrat F., Carsley J. (2007). Forming of AA5182-O and AA5754-O at elevated temperatures using coupled thermo-mechanical finite element models. Int. J. Plast..

[22] Lei C., Wang Q., Tang H., Liu T., Li Z., Jiang H., Wang K., Ebrahimi M., Ding W. (2021). Hot deformation constitutive model and processing maps of homogenized Al–5Mg–3Zn–1Cu alloy. J. Mater. Res. Technol..

[23] Lei C., Wang Q., Ebrahimi M., Li D., Tang H., Zhang N., Cai H. (2023). Hot deformation behavior and processing maps of an as-cast Al-5Mg-3Zn-1Cu (wt%) alloy. Materials.

[24] Wu H., Wen S.P., Huang H., Gao K.Y., Wu X.L., Wang W., Nie Z.R. (2016). Hot deformation behavior and processing map of a new type Al-Zn-Mg-Er-Zr alloy. J. Alloys Compd..

[25] Wei T., Wang Y., Tang Z., Xiao S. (2021). The constitutive modeling and processing map of homogenized Al-Mg-Si-Cu-Zn alloy. Mater. Today Commun..

[26] Heidarzadeh A., Mohammadzadeh R., Jafarian H.R., Pruncu C.I., Simar A. (2022). Role of geometrically necessary dislocations on mechanical properties of friction stir welded single-phase copper with medium stacking fault energy. J. Mater. Res. Technol..

[27] Liu S., Pan Q., Li H., Huang Z., Li K., He X., Li X. (2019). Characterization of hot deformation behavior and constitutive modeling of Al–Mg–Si–Mn–Cr alloy. J. Mater. Sci..

[28] Clayton D. (2005). Dynamic plasticity and fracture in high density polycrystals: Constitutive modeling and numerical simulation. J. Mech. Phys. Solids.

[29] Cheong K.S., Busso E.P. (2004). Discrete dislocation density modelling of single phase FCC polycrystal aggregates. Acta Mater..

[30] Asaro R., Needleman A. (1985). Texture development and strain hardening in rate dependent polycrystals. Acta Metall..

[31] Laasraoui A., Jonas J.J. (1991). Prediction of steel flow stresses at high temperatures and strain rates. Metall. Trans. A.

[32] Chen B., Tian X., Li X., Lu C. (2014). Hot deformation behavior and processing maps of 2099 Al-Li alloy. J. Mater. Eng. Perform..

[33] Zheng X., Luo A.A., Sachdev K., Ding W. (2012). Plastic flow behavior of a high-strength magnesium alloy NZ30K. Mater. Sci. Eng. A.

[34] Meng Q., Bai C., Xu D. (2018). Flow behavior and processing map for hot deformation of ATI425 titanium alloy. J. Mater. Sci. Technol..

[35] Cheng C., Ji Y., Guo X., Abd El-Aty A. (2024). Coupling theoretical analysis and FE framework for revealing the size effect on the deformation characteristics of 304 stainless steel microtubes manufactured via free-bending forming technology. CIRP J. Manuf. Sci. Technol..

[36] Zener C., Hollomon J.H. (1944). Effect of strain rate upon plastic flow of steel. J. Appl. Phys..

[37] Shi H.M. (1997). Constitutive equations for high temperature flow stress of aluminium alloys. Mater. Sci. Technol..

[38] Lin Y.C., Chen M., Zhong J. (2008). Constitutive modeling for elevated temperature flow behavior of 42CrMo steel. Comput. Mater. Sci..

[39] Samantaray D., Mandal S., Bhaduri A.K. (2011). Analysis and mathematical modelling of elevated temperature flow behaviour of austenitic stainless steels. Mater. Sci. Eng. A.

[40] Samantaray D., Mandal S., Borah U., Bhaduri A.K. (2009). A thermo-viscoplastic constitutive model to predict elevated-temperature flow behaviour in a titanium-modified austenitic stainless steel. Mater. Sci. Eng. A.

[41] Cheng C., Guo J., Abd El-Aty A., Guo X. (2024). Approach Based on Response-Surface Method to Optimize Lining of Dies Used in 3D Free-Bending Forming Technology. Chin. J. Mech. Eng..

[42] Zhao P., Cheng C., Abd El-Aty A., Tao J., Guo X., Ji Y. (2024). Multiscale framework-based crystal plasticity modeling and texture evolution of the deformation behavior of AISI 304 stainless steel microtubes manufactured through 3D-FBF technology. Sustain. Mater. Technol..

[43] Elshaer R.N., El-Aty A.A., Sayed E.M., Barakat A.F., Sobh A.S. (2024). Optimization of machining parameters for turning operation of heat-treated Ti-6Al-3Mo-2Nb-2Sn-2Zr-1.5Cr alloy by Taguchi method. Sci. Rep..

[44] Samantaray D., Mandal S., Bhaduri A.K. (2010). Constitutive analysis to predict high-temperature flow stress in modified 9Cr-1Mo (P91) steel. Mater. Des..

[45] Ou L., Zheng Z., Nie Y., Jian H. (2015). Hot deformation behavior of 2060 alloy. J. Alloys Compd..

[46] Yaich M., Gavrus A. (2020). New phenomenological material constitutive models for the description of the Ti_6_Al_4_V titanium alloy behavior under static and dynamic loadings. Proc. Manuf..

[47] Ma L., Wan M., Li W., Shao J., Bai X., Zhang J. (2021). Superplastic deformation mechanical behavior and constitutive modelling of a near-α titanium alloy TNW700 sheet. Mater. Sci. Eng. A.

[48] Hrot A., Beaker M. (2012). Determination of Johnson–Cook parameters from machining simulations. Comput. Mater. Sci..

[49] Qiao L., Zhu J. (2022). Constitutive modeling of hot deformation behavior of AlCrFeNi multi-component alloy. Vacuum.

[50] Wang H., Qin G., Li C. (2022). A modified Arrhenius constitutive model of 2219-O aluminum alloy based on hot compression with simulation verification. J. Mater. Res. Technol..

[51] Hu S., Cheng C., Guo X., Abd EI-Aty A. (2024). Investigation of tube free bending forming process based-parallel mechanism to manufacture 304 stainless steel spiral tubes. Manuf. Lett..

[52] Shokry A., Gowid S., Youssef S.S. (2022). Modeling the flow behavior of Haynes 214 superalloy during hot deformation using mathematical and artificial intelligence-based models. Mater. Today Commun..

[53] Shokry A. (2024). Modified Fields-Backofen and Zerilli-Armstrong constitutive models to predict the hot deformation behavior in titanium-based alloys. Sci. Rep..

[54] Abd El-Aty A., Xu Y., Hou Y., Zhang S.H., Ha S., Xia L., Shokry A. (2024). Modelling the flow behaviour of Al alloy sheets at elevated temperatures using a modified Zerilli–Armstrong model and phenomenological-based constitutive models. Materials.

[55] Jia W., Xu S., Le Q., Fu L., Ma L., Tang Y. (2016). Modified Fields-Backofen model for constitutive behavior of as-cast AZ31B magnesium alloy during hot deformation. Mater. Des..

[56] Zhao P., Cheng C., Abd El-Aty A., Tao J., Guo X., Ji Y. (2024). Integrating crystal plasticity and experimentation for investigating the size effect on the deformation characteristics of AISI304 microtubes manufactured by free bending technology. Manuf. Lett..

[57] Kumar S., Karmakar A., Nath S.K. (2021). Construction of hot deformation processing maps for 9Cr-1Mo steel through conventional and ANN approach. Mater. Today Commun..

[58] Samantaray D., Mandal S., Bhaduri A.K. (2009). A comparative study on Johnson–Cook, modified Zerilli–Armstrong and Arrhenius-type constitutive models to predict elevated temperature flow behaviour in modified 9Cr–1Mo steel. Comput. Mater. Sci..

[59] Johnson G.R., Cook W.H. A constitutive model and data for metals subjected to large strains, high strain rates and high temperatures. Proceedings of the 7th International Symposium on Ballistics.

[60] Lin Y.C., Chen X.-M., Liu G. (2010). A modified Johnson–Cook model for tensile behaviors of typical high-strength alloy steel. Mater. Sci. Eng. A.

[61] Akbari Z., Mirzadeh H., Cabrera J.-M. (2015). Material design. Mater. Des..

[62] Chen L., Zhao G., Yu J. (2015). Material design. Mater. Des..

[63] Mirzadeh H. (2015). Mechanical properties of materials. Mech. Mater..

[64] He A., Xie G., Zhang H., Wang X. (2014). Material design. Mater. Des..

[65] Lin Y.C., Chen X.-M. (2010). Computational material science. Comput. Mater. Sci..

[66] Li H.Y., Wang X.F., Duan J.Y., Liu J.J. (2013). Material science and engineering. Mater. Sci. Eng. A.

[67] Lin Y.C., Li L.-T., Fu Y.-X., Jiang Y.-Q. (2012). Journal of material science. J. Mater. Sci..

[68] Abbasi-Bani A., Zarei-Hanzaki A., Pishbin M.H., Haghdadi N. (2014). Mechanical properties of materials. Mech. Mater..

[69] Song W., Ning J., Mao X., Tang H. (2013). Material science and engineering. Mater. Sci. Eng. A.

[70] Ghosh A., Elasheri A., Parson N., Chen X.-G. (2024). Hot deformation behavior and processing maps for an Al-Mg-Si-Zr-Mn alloy. J. Alloys Metall. Syst..

[71] Li T., Lu Y., Li Z., Wang T., Li T. (2022). Hot deformation behavior and microstructure evolution of non-equimolar Ti_2_ZrHfV_0.5_Ta_0.2_ refractory high-entropy alloy. Intermetallics.

[72] Li X., Le Q., Li D., Wang P., Jin P., Cheng C., Cheng X., Ren L. (2021). Hot tensile deformation behavior of extruded LAZ532 alloy with heterostructure. Mater. Sci. Eng. A.

[73] Che B., Lu L., Kang W., Luo J., Ma M., Liu L. (2021). Hot deformation behavior and processing map of a new type Mg-6Zn-1Gd-1Er alloy. J. Alloys Compd..

[74] Shokry A., Ståhle P. (2015). A methodology for using Kalman filter to determine material parameters from uncertain measurements. Mater. Discov..

[75] Chen Y., Ye L., Dong H. (2023). Lightweight 3D carbon fibre reinforced composite lattice structures of high thermal-dimensional stability. Compos. Struct..

[76] Chen Y., Xu C., Wang C.H., Bilek M.M.M., Cheng X. (2022). An effective method to optimise plasma immersion ion implantation: Sensitivity analysis and design based on low-density polyethylene. Plasma Process. Polym..

